# Illness Experiences of Young Women With Rheumatoid Arthritis in South Korea

**DOI:** 10.1111/hex.70771

**Published:** 2026-07-18

**Authors:** Wonhee Baek, Hyunjung Doo

**Affiliations:** ^1^ College of Nursing Gyeongsang National University Jinju Gyeongnam South Korea; ^2^ Department of Nursing, College of Health Sciences Kyungnam University Changwon Gyeongnam South Korea

**Keywords:** rheumatoid arthritis, self‐disclosure, social stigma, women, young adult

## Abstract

**Background:**

Rheumatoid arthritis affects young women during critical life‐course stages, requiring ongoing adaptation to illness‐related uncertainty while managing social roles and expectations. Women in their 20s to 40s face distinctive challenges related to illness disclosure, stigma and life‐course tasks, particularly around childbirth and caregiving roles.

**Objective:**

This qualitative study explored the illness experiences of women with rheumatoid arthritis in their 20s to 40s and examined how they construct everyday normality amid uncertainty and life‐course tasks.

**Design:**

Colaizzi's descriptive phenomenological method.

**Setting and Participants:**

Semi‐structured in‐depth interviews were conducted with 20 women aged 20–49 years who met the 2010 American College of Rheumatology/European Alliance of Associations for Rheumatology classification criteria. The mean interview duration was 58 min. Colaizzi's seven‐step phenomenological analysis was applied; coding consistency was checked (Cohen's kappa = 0.90).

**Results:**

From 529 meaning units, 42 subthemes, 12 core themes and 5 theme clusters were derived, with an overarching theme of ‘Constructing normality between uncertainty and life‐course tasks’. Participants employed differentiated disclosure strategies, transitioning from concealment to selective disclosure around childbirth. Two novel concepts were identified: ‘forced disclosure’, wherein illness was unintentionally exposed due to structural factors, and ‘legitimised vulnerability’, wherein social approval for help‐seeking was obtained through the maternal role.

**Discussion:**

Young Korean women with rheumatoid arthritis reported experiencing patterns of diagnostic delay, shifts in disclosure strategies with childbirth as the turning point, and maternal role conflict. Participants did not experience disclosure as a purely individual decision. Instead, they feared that disclosure would lead to being devalued as ‘a sick person’ or ‘a weak person’, which shaped their disclosure strategies.

**Conclusions:**

Childbirth serves as a turning point in illness disclosure strategies, highlighting the need for nursing interventions tailored to life‐course tasks.

**Lived Experience or Public Contribution:**

A registered nurse who is herself living with rheumatoid arthritis contributed to developing and refining the interview questions during the study design phase. She also provided limited input during analysis by helping to confirm the meaning of some participant statements, enhancing the study's credibility and relevance to the experiences of young women with RA.

## 1 Introduction

Rheumatoid arthritis (RA) is a long‐term autoimmune disease marked by persistent inflammation of synovial joints, with both incidence and prevalence about two to three times higher in women than in men [1, 2]. The burden of RA among women of childbearing age is rising: globally, incident RA cases in women aged 15–49 increased by 68% between 1990 and 2021 [3], and in South Korea, the incidence of seropositive RA in women aged 20–44 rose from 21.0 to 28.4 per 100,000 person‐years over 2011–2016 [4].

Because RA symptoms are not readily visible, it is often described as an ‘invisible illness’, and patients' difficulties are frequently underestimated by others [5, 6]. A systematic review showed that younger age and female sex are independently associated with higher anxiety [7], indicating that young women with RA may face compounded vulnerability.

Young women with RA often manage long‐term disease during a life stage characterised by developmental tasks such as career development, marriage, pregnancy, childbirth and childrearing. These overlapping demands create challenges that differ qualitatively from those faced by older individuals. Birru Talabi et al. reported that 58% of women with inflammatory arthritis modified their family planning because of the disease, and 85% expressed concerns about their ability to care for children [8]. Fear of teratogenic medications contributes to anxiety about not being able to have a healthy child, whereas physical limitations lead to guilt about not being able to be a good mother [9, 10]. However, substantial gaps remain in information‐seeking and healthcare utilisation related to pregnancy decisions [10], emphasising the need for preconception counselling and multidisciplinary approaches [11].

Qualitative research on the illness experiences of RA patients remains limited in two aspects. First, since Hwang et al.'s study, no phenomenological studies have examined the life‐cycle experiences of young women in South Korea in over 20 years [12], except for Kim and Sung's study on quality of life using structural equation modelling [13]. Second, existing meta‐syntheses and mega‐ethnographies have included only Western studies [6, 14], highlighting the need to examine ‘cultural nuances’ in non‐Western contexts. In Korea, strong social and cultural expectations surrounding traditional maternal roles, including childbearing [15], may place distinctive pressures on young women with RA as they navigate illness disclosure and negotiate their identities as wives and mothers. This suggests that patterns of illness disclosure and identity negotiation may differ from those observed in Western individualist cultures.

Bury's concept of ‘biographical disruption’ has been central to theoretical understandings of long‐term illness [16]. Originally developed through work with individuals with RA, the concept is directly relevant to the present study, as it captures how diagnosis necessitates the reconstruction of self‐identity and life trajectory. Rather than being superseded, biographical disruption has continued to be applied and developed in recent qualitative research on long‐term illness, where it is increasingly understood as non‐linear and mediated by life stage, gender and culture [17], including studies of young women in East Asian collectivist contexts [18, 19].

Goffman's stigma management theory explains how individuals with invisible illnesses manage information through strategies such as ‘passing’, ‘selective disclosure’ and ‘voluntary disclosure’ [20]. Goffman's framework remains foundational to later models of health‐related stigma and concealable stigmatised identities [21, 22] Parsons' sick role is invoked here only as a contrast baseline; its assumption of temporary exemption and expected recovery has been widely criticised as inadequate for long‐term illness and has since been reworked within social‐constructivist and candidacy‐based accounts, on which the present analysis draws in preference to the sick role [23, 24]. However, these theories have been largely developed in individualist cultural contexts, and identity negotiation within collectivist cultures remains insufficiently explored, which this study addresses by extending these established frameworks to the Korean context.

Accordingly, this study explored the illness experiences of young women with RA using Colaizzi's descriptive phenomenological method, which is suited for capturing experiential patterns in the Korean context without reliance on assumptions embedded in Western theories. Through an in‐depth understanding of these experiences, this study sought to provide a foundation for developing life‐cycle‐tailored nursing interventions and contribute to the advancement of nursing knowledge. The specific research questions were:

1. What is the essential structure of illness experiences among young women with RA?
2. How do illness experiences differ across life stages (20s, 30s and 40s)?
3. What distinctive experiential patterns emerge within the Korean cultural context?

## 2 Materials and Methods

### 2.1 Design

This qualitative study employed Colaizzi's phenomenological methodology [25]. Colaizzi's method is grounded in Husserl's transcendental phenomenology, with bracketing—the suspension of the researcher's preconceptions—as its core principle [26]. Reporting followed the Consolidated Criteria for Reporting Qualitative Research (COREQ) [27].

### 2.2 Participants

Purposive and snowball sampling [28, 29] were combined to recruit 20 women with RA aged 20–49 (Table 1). Maximum variation sampling was used to ensure diversity in disease activity, disease duration, marital status and employment status [29]. Inclusion criteria were: (1) confirmed diagnosis based on the 2010 American College of Rheumatology/European Alliance of Associations for Rheumatology (ACR/EULAR) classification criteria, (2) initial diagnosis between ages 18–39 years, (3) currently aged 20–49 years and (4) a minimum of 6 months since diagnosis.

**Table 1:** Participant characteristics (*N* = 20).

ID	Age/marital status	Children	Age at Dx	Diagnostic delay (months)	Disease duration (years)	Occupation	Primary medications	Serostatus
P01	41/Married	1	36	12	5	Office worker	csDMARD(MTX), NSAID	Positive
P02	48/Married	2	36	18	12	Self‐employed	bDMARD inhibitor, Steroid	Positive
P03	48/Married		39	9	9	Self‐employed	csDMARD(MTX), Steroid	Positive
P04	26/Unmarried		18	9	8	Professional	csDMARD(MTX), Steroid	Positive
P05	35/Married	1	29	12	6	Office worker	bDMARD, Analgesics	Positive
P06	27/Unmarried		24	6	3	Office worker	csDMARD(MTX), Steroid	Positive
P07	33/Unmarried		29	6	4	Office worker	Steroid	Negative
P08	24/Unmarried		22	12	2	Office worker	NSAID	Positive
P09	36/Married	1	26	10	10	Professional	csDMARD(MTX), Steroid	Positive
P10	40/Married	1	33	12	7	Freelancer	csDMARD(SSZ), Analgesics	Positive
P11	47/Married	1	35	24	12	Self‐employed	csDMARD(MTX), Steroid	Positive
P12	35/Married	1	29	12	6	Office worker	NSAID	Positive
P13	36/Unmarried		33	12	3	Professional	Steroid	Positive
P14	38/Married	2	29	12	9	Self‐employed	csDMARD(SSZ), Analgesics	Positive
P15	43/Married	1	36	12	7	Production worker	csDMARD(SSZ), Steroid	Positive
P16	33/Unmarried		32	12	1	Office worker	csDMARD(MTX), Steroid	Positive
P17	48/Married	1	36	12	12	Office worker	csDMARD(MTX), Steroid	Positive
P18	28/Unmarried		26	12	2	Office worker	csDMARD(MTX), Steroid	Positive
P19	36/Married	1	34	12	2	Freelancer	DMARD	Positive
P20	29/Unmarried		25	9	4	Professional	DMARD	Negative

Abbreviations: bDMARD, biologic disease‐modifying antirheumatic drug; csDMARD, conventional synthetic disease‐modifying antirheumatic drug; Dx, diagnosis; MTX, methotrexate; NSAID, nonsteroidal anti‐inflammatory drug; SSZ, sulfasalazine.

The final sample of 20 was not fixed in advance but was determined using the principle of information power under which a sample carrying more information relevant to the study aim requires fewer participants [30]. Given the narrow focus of the study, the specificity of the sample, and the grounding of the analysis in established theory, the data from these 20 participants were judged to carry sufficient information power for the analytic aim, with adequate depth and richness to address the research question. Participants were recruited through notices posted online and circulated among acquaintances. Each individual in the study contacted the research team on their own initiative to express interest in taking part. Because recruitment rested on this self‐selection rather than on the researchers approaching potential participants, no eligible person who reached out subsequently declined to participate.

### 2.3 Data Collection

Following ethical approval, a recruitment advertisement was posted. Recruitment, enrolment and individual in‐depth interviews were conducted on a rolling basis between 15 November 2025 and 17 January 2026. After seeing the recruitment advertisement or hearing about the study from a referrer, individuals who were willing to take part contacted the researcher by email or telephone; the researcher then arranged and conducted in‐depth interviews with those who had expressed their willingness to participate. Data collection and analysis proceeded concurrently and iteratively, and the information power sufficiency was confirmed in January 2026; participant validation (returning the findings to participants, the seventh step of Colaizzi's method) was carried out during this period as an integral part of the iterative process rather than as a separate final stage. Interviews lasted an average of 58 min (range: 45–90 min) and were held either in person (10 participants) or by telephone (10 participants), depending on participant preference. Telephone interviewing is an established qualitative method that yields data comparable to face‐to‐face interviews and may facilitate disclosure of sensitive or stigmatised experiences [31, 32]. For seven participants, a second interview was conducted after reviewing the first interview transcript, in order to clarify the meaning of their statements in greater depth; these follow‐up interviews formed part of data collection and were distinct from the validation of the findings (the seventh step of Colaizzi's method) described in the Data Analysis and Rigour sections.

The semi‐structured interview guide was reviewed and revised by two nursing professors with qualitative research expertise. A registered nurse who is herself living with RA also contributed, as a non‐author patient advisor, to the development of the interview questions. Pilot interviews were conducted with two individuals with RA to assess question clarity and appropriateness. Their feedback indicated that the question on ‘the impact of the disease on daily life’ was overly broad; accordingly, it was divided into ‘work life’, ‘home life’ and ‘social relationships’. The sequence of pregnancy‐ and childbirth‐related questions was also adjusted to reduce psychological burden associated with sensitive topics.

The interview guide included an opening question (‘Please feel free to talk about your experience living with an RA diagnosis’), followed by questions on diagnosis experience, daily life impact, pregnancy, childbirth, childrearing (if applicable), social relationships and illness disclosure, coping and support and closing questions.

### 2.4 Data Analysis

**Figure 1:**
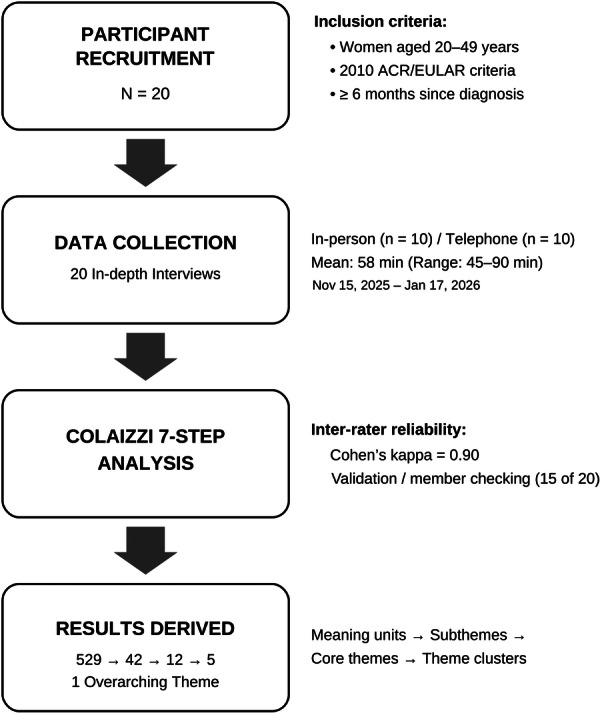
Study process.

Colaizzi's seven‐step phenomenological analysis involved: (1) repeated reading of transcripts, (2) extraction of significant statements (529), (3) formulation of meanings, (4) organisation of themes (42 subthemes), (5) integration into theme clusters (12 core themes and 5 theme clusters), (6) exhaustive description and (7) validating the findings by returning the exhaustive description to participants [25]. The principal investigator coded all transcripts using MAXQDA 2020 (VERBI Software, Berlin, Germany). To enhance coding credibility, two researchers who were not part of the author team independently coded eight randomly selected transcripts (40%); the level of coding agreement (Cohen's kappa) was 0.90. Quotations were selected to represent core thematic meanings and balance participants' voices. Interviews were transcribed by the principal investigator, anonymised using codes, and stored in encrypted cloud storage. Figure 1 illustrates the overall study process.

### 2.5 Rigour

Lincoln and Guba's trustworthiness criteria were applied [33]. For credibility, written analysis results were shared with all 20 participants for validation, the seventh step of Colaizzi's method (member checking); five did not respond, and the remaining 15 confirmed the findings, of whom 12 agreed that their experiences were accurately reflected while three requested minor adjustments to wording nuance, which were incorporated into the final analysis. This procedure was one of several strategies used to enhance trustworthiness, alongside peer debriefing, double coding and bracketing. Peer debriefing was conducted with two nursing doctoral researchers to verify analytical coherence. Additionally, the patient advisor provided limited input during analysis by helping confirm the meaning of some participant statements. For confirmability, bracketing was systematically implemented. The principal investigator, a nursing researcher with 5 years of clinical experience and over 10 years of research experience in women's health, has conducted research on RA. This ongoing research engagement with the RA population provided contextual understanding of disease‐specific challenges, while bracketing was rigorously applied to remain open to emergent findings. The investigator shared no prior relationship with the participants. Transcription and primary coding were carried out by the principal investigator, and coding consistency was independently checked by two colleagues who are acknowledged below. Preunderstanding was documented in a reflective journal before data collection, including assumptions that young women with RA would delay childbirth and individuals with invisible illnesses would be suspected of symptom exaggeration. During analysis, participants actively pursued childbirth and strategic information control using illness invisibility was observed, contradicting initial assumptions.

### 2.6 Ethical Considerations

This study received approval from the Institutional Review Board of Gyeongsang National University (IRB No. GIRB‐G25‐NY‐0126; Approval date: 5 November 2025). Written consent was obtained after participants were informed of the study purpose, procedures, voluntary participation and withdrawal rights, and confidentiality protections, with separate consent for interview recording. Because interviews addressed sensitive topics such as pregnancy, childbirth and workplace discrimination, interviews were paused when emotional discomfort was expressed, rest was offered and information on professional psychological counselling services was provided when necessary. Although these procedures were in place, no participant experienced distress severe enough to require interview suspension, and none requested or required referral to professional counselling services.

## 3 Results

**Figure 2:**
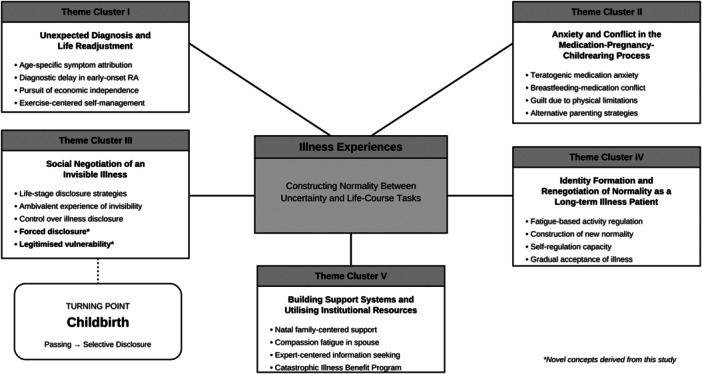
Thematic structure of illness experiences among young women with rheumatoid arthritis. The overarching theme ‘Constructing normality between uncertainty and life‐course tasks’ encompasses five theme clusters. Theme Cluster III (Social Negotiation of an Invisible Illness) is highlighted because it contains two novel concepts derived in this study. Childbirth emerged as a turning point that shifted disclosure strategies from passing (concealment) to selective disclosure. *Novel concepts derived in this study.

Colaizzi's phenomenological analysis yielded 529 meaning units, which were grouped into 42 subthemes, 12 core themes and 5 theme clusters (Table 2). The overarching theme was ‘Constructing normality between uncertainty and life‐course tasks’. Figure 2 illustrates the thematic structure.

**Table 2:** Thematic structure of illness experiences among young women with rheumatoid arthritis.

Theme cluster	Core theme	Subtheme	*n*
I. Unexpected diagnosis and life readjustment	1. Diagnostic delay in early‐onset RA	1.1 Age‐specific initial attribution of symptoms	23
		1.2 Diagnostic delay due to a lack of autoimmune disease awareness	20
		1.3 Initial uncertainty and lack of empathy after diagnosis	12
	2. Medication stabilisation and adaptation after diagnosis	2.1 Relief from acute symptom pain reduction	16
		2.2 Difficulty during optimal medication search	19
		2.3 Pursuit of economic independence among unmarried women	6
		2.4 Exercise‐centred self‐management	15
II. Anxiety and conflict in the medication–pregnancy–childrearing process	3. Fear of teratogenic medications and pregnancy decisions	3.1 Anxiety about teratogenic medications	16
		3.2 Burden of medication discontinuation for pregnancy preparation	14
		3.3 Anxiety about foetal health during pregnancy	12
		3.4 Deferral of family planning due to illness	8
	4. Maternal role constraints and guilt	4.1 Guilt about giving up breastfeeding	10
		4.2 Conflict between continuing breastfeeding and resuming medication	8
		4.3 Guilt due to physical limitations	13
		4.4 Seeking alternative parenting strategies	9
III. Social negotiation of an invisible illness	5. Childbirth bringing a turning point in disclosure strategies	5.1 Participants aged 20s–30s without children: Active concealment	13
		5.2 Participants aged 20s–30s with children: Selective disclosure	13
		5.3 Participants in their 40s: Open disclosure in the name of ageing	10
	6. Ambivalent outcomes of invisibility	6.1 Facilitation of stigma avoidance along with the receipt of insufficient empathy	18
		6.2 Normalisation of long‐term pain and fatigue	14
		6.3 Lack of social awareness about rare diseases	6
		6.4 Discrepancy between inflammatory markers and perceived symptoms	17
	7. Control over illness disclosure	7.1 Involuntary exposure led to forced disclosures	14
		7.2 Polarised disclosure experiences based on workplace environment	13
		7.3 Concerns about discrimination and career disruption	20
IV. Identity formation and renegotiation of normality as a long‐term illness patient	8. Fatigue‐based activity regulation strategies	8.1 Energy distribution at home	14
		8.2 Use of assistive devices at work	13
		8.3 Utilisation of smart home appliances	12
		8.4 Changes in job type and work pattern	6
		8.5 Symptom concealment and job retention	7
	9. Construction of a new normal	9.1 Sense of disconnection from pre‐illness self	15
		9.2 Gradual acceptance of illness	14
		9.3 Designing life differently	12
		9.4 Acquisition of self‐regulation capacity	11
V. Building support systems and utilising institutional resources	10. Distributed burden through family support	10.1 Natal family‐centred caregiving support	18
		10.2 Compassion fatigue in spouses	10
	11. Expert‐centred information‐seeking	11.1 Limited reliable information	11
		11.2 Recognition of commercial bias in online information	9
		11.3 Rational reliance on medical professionals	14
		11.4 Limited utilisation of patient communities	8
	12. Catastrophic Illness Benefit Programme and institutional gaps	12.1 Reduced economic burden through the Catastrophic Illness Benefit Programme	10
		12.2 Institutional exclusion of seronegative RA	6
Total	12	42	529

*Note: n* indicates the frequency of meaning units extracted for each subtheme.

### 3.1 Theme Cluster I: Unexpected Diagnosis and Life Readjustment

Most participants reported pain in the fingers and wrists, about half also reported pain in the soles of the feet and shoulders, and some reported knee pain, reflecting the multi‐joint nature of RA; the wrists, however, were most prominent in their accounts because hand and wrist function was central to childcare, household work and office work. Participants attributed symptoms occurring at a young age to life‐stage‐related causes, which resulted in delayed diagnosis. Unmarried participants in their 20s and 30s attributed symptoms to excessive computer use, post‐childbirth participants to postpartum musculoskeletal strain or childcare‐related fatigue, and participants in their 40s to ageing.

My wrist hurt, so I thought it was from using the computer too much. I assumed I was not exercising enough. I never imagined a young person could have arthritis.

(Participant 13, 36 years, unmarried, professional, 3 years since diagnosis)

Everyone says wrist pain after childbirth is common. All the other mothers around me said so. I assumed it was due to inadequate postpartum self‐care.

(Participant 19, 36 years, married, 2 years post‐childbirth, freelancer)

After diagnosis, participants experienced medication side effects and limited efficacy while attempting to identify effective treatment. Unmarried participants perceived that RA could undermine marriage prospects and therefore pursued economic independence.

### 3.2 Theme Cluster II: Anxiety and Conflict in the Medication–Pregnancy–Childrearing Process

Participants reported fear of teratogenic medications such as methotrexate (MTX), the burden of discontinuing treatment for pregnancy, and persistent anxiety about foetal health. In particular, medication use was perceived not only as medical information but also as sensitive information that could invite stigma as ‘a woman taking drugs harmful to the foetus’.

They say rheumatoid arthritis medication is really bad for the foetus. They tell you it's fine if you stop taking it a few months before getting pregnant, but who knows if it stays in your body? I think this way, so wouldn't others think the same? That's why I hesitate to tell people. I just hope others don't find out.

(Participant 4, 26 years, unmarried, professional, 8 years since diagnosis)

I'm worried about how much worse I'll get while not taking medication during pregnancy preparation. There could be a lot of joint deformity too. I don't know what to do.

(Participant 6, 27 years, unmarried, office worker, 3 years since diagnosis)

Participants felt guilty about being unable to care adequately for their children because of joint pain and fatigue, and were conflicted about breastfeeding versus resuming medication. They developed alternative parenting strategies to compensate for physical limitations.

My child wants to be held, but my wrist hurts so I can't hold them. At those times, I really feel sorry … like I'm not qualified to be a mother.

(Participant 10, 40 years, married, 7 years post‐childbirth, freelancer)

Instead of holding, I read a lot of books beside them. I can still read books. Even if the baby can't understand yet, I talk to them a lot. That's my own way.

(Participant 1, 41 years, married, 5 years post‐childbirth, office worker)

### 3.3 Theme Cluster III: Social Negotiation of an Invisible Illness

Disclosure strategies varied across life stages (Table 3). Unmarried participants in their 20s and 30s concealed their illness to avoid negative effects on marriage prospects, whereas post‐childbirth participants disclosed selectively to obtain childcare support, and participants in their 40s disclosed joint pain as a normative aspect of ageing (‘everyone experiences pain as they get older’).

Even with my boyfriend, I never said it was rheumatoid arthritis—just that I had ‘some autoimmune condition.’ With his family and others, I said nothing, or at most called it a kind of autoimmune disease. Naming RA specifically would bring up the medication and might threaten marriage, and that frightened me.

(Participant 20, 29 years, unmarried, professional, 4 years since diagnosis)

Before having a child, when I said I have rheumatoid arthritis, people would react like ‘What kind of arthritis for someone so young?’ But after giving birth, when I said, ‘My wrist got worse from taking care of the baby,’ everyone said, ‘That can happen.’ It's the same pain, but when I connect it to the child, people accept it differently.

(Participant 14, 38 years, married, 9 years post‐childbirth, self‐employed)

**Table 3:** Differentiated characteristics of illness experience by age group (*N* = 20).

Dimension	20s (*n* = 5)	30s (*n* = 8)	40s (*n* = 7)
Initial symptom attribution	Overwork, excessive computer use, stress	Postpartum symptoms, childcare fatigue (married)/Work stress (unmarried)	Natural aging process
Disclosure strategy	Active concealment (e.g., disguising as carpal tunnel syndrome)	Transition to selective disclosure (post‐childbirth)/Persistent concealment (unmarried)	Open disclosure utilising age norms
Primary life‐task concerns	Career development, marriage prospects, economic independence	Pregnancy/childbirth decisions, work‐family balance, family planning	Disease progression management, institutional resource utilisation
Support system priorities	Online patient communities, peer support	Natal family‐centred caregiving support, spousal sharing	Long‐term trust relationship with medical professionals
Self‐management strategies	Exercise‐centred management (swimming, yoga)	Energy distribution, smart appliance utilisation, household priority adjustment	Medication self‐adjustment capacity, body signal interpretation
Key stigma management issues	‘Healthy spouse’ image maintenance, avoiding marriage market disadvantage	Meeting maternal role expectations, controlling teratogenic medication information	Fewer stigma management concerns due to age‐normative framing of long‐term illness

*Note:* Age groups are based on age at the time of interview. The 30s group includes both unmarried (*n* = 3) and married (*n* = 5) participants, with subgroup differences present.

The workplace environment, alongside childbirth, shaped disclosure strategies. Participant 13 (age 36, unmarried, professional) worked in a setting where women in their 30s and 40s predominated, and many colleagues used long‐term medication for long‐term conditions such as thyroid disorders and hypertension. In this context, she disclosed her illness soon after diagnosis despite having no childbirth experience and reported receiving empathy and support from colleagues. By contrast, Participant 7 (age 33, unmarried, office worker) was diagnosed with seronegative RA and excluded from institutional benefits under the Catastrophic Illness Benefit Programme. This exclusion fostered a perception of not being recognised as a ‘legitimate patient’, creating a stronger barrier to disclosure.

The invisibility of RA produced ambivalent outcomes: while it facilitated stigma avoidance, it also hindered the receipt of empathy. Participants further described ‘medical invisibility’, reporting pain and fatigue despite objective test values remaining within the normal range.

I look fine on the outside, so even when I say I'm in pain, they don't believe me. ‘You look healthy, though?’ That's the hardest thing to hear. So, once I deliberately showed them my swollen finger.

(Participant 18, 28 years, unmarried, office worker, 2 years since diagnosis)

A newly identified phenomenon in this study was ‘forced disclosure’. Participants experienced unintended illness exposure through regular hospital visits or sudden symptom flares.

I take sick leave or annual leave to go to the hospital, but I end up having to tell my supervisor. But it gets revealed against my will that way. I can't even ask them to keep it a secret.

(Participant 3, 48 years, married, self‐employed, 9 years since diagnosis)

Reactions following illness disclosure varied sharply based on workplace environment, and participants expressed concerns about discrimination and career disruption, including the worry that disclosure might foreclose opportunities to have their competence fairly evaluated at work.

### 3.4 Theme Cluster IV: Identity Formation and Renegotiation of Normality as a Long‐Term Illness Patient

To manage limited energy, participants reprioritised household activities and used smart home appliances such as robot vacuums, dishwashers and clothes dryers. They also adopted proactive energy allocation strategies, including completing work in advance on days when they felt well in anticipation of symptom flares.

A lot of people use smart appliances these days—robot vacuums, dishwashers, clothes dryers. They're a bit pricey, but I bought them on purpose to put less strain on my joints. Even if it costs money, I try to manage the housework smartly, for the sake of my own body.

(Participant 12, 35 years, married, 6 years since diagnosis, office worker)

If I clean today, I have to rest tomorrow. I can't do everything at once. So, Monday is cleaning, Wednesday is laundry—I divide it up like that.

(Participant 5, 35 years, married, 6 years post‐childbirth, office worker)

Participants with longer disease duration developed self‐management competency, accepting the illness as part of life and adjusting medication dosage within the range prescribed by their healthcare provider based on symptoms.

After living with it for a few years, even when taking medication, there are days when the pain suddenly gets worse, or I swell up. On those days, I adjust the amount of the medication the doctor prescribed to take as needed. I can manage myself now.

(Participant 7, 33 years, unmarried, office worker, 4 years since diagnosis)

### 3.5 Theme Cluster V: Building Support Systems and Utilising Institutional Resources

Participants primarily received practical care support from their mothers and maternal family members, and some also drew on free counselling services at community centres to cope with the emotional difficulty of accepting the diagnosis. Participants also experienced difficulties with information‐seeking because of limited reliable resources for this rare disease and critically recognised commercial bias in online information, choosing to rely on professional healthcare providers as a rational strategy. Most participants (18) reported reduced healthcare costs through the Catastrophic Illness Benefit Programme, noting that this support was decisive for continuing treatment given the high cost of biological agents (over 1 million Korean won, approximately USD 750 per month). By contrast, participants with seronegative RA (2) reported inequity resulting from exclusion from institutional benefits despite comparable symptoms and treatment.

At first the pain was so severe I couldn't think of anything else. But once the pain eased, that was when the mental side hit me—having to live with this for the rest of my life, taking medication forever… coming to terms with becoming an RA patient was really hard. By chance I found free counselling at the community centre and received it for about a year; it really helped.

(Participant 11, 47 years, married, self‐employed, 12 years since diagnosis)

I moved right next to my older sister's home. My mother worries a great deal and comes by often, and my sister and brother‐in‐law take my child to the playground and give rides.

(Participant 9, 36 years, married, professional, 10 years since diagnosis)

Since I got the Catastrophic Illness Benefit, my medication costs were reduced to one‐tenth. Biological agents originally cost over a million won a month—I couldn't have received treatment without this.

(Participant 2, 48 years, married, self‐employed, 12 years since diagnosis)

## 4 Discussion

Young Korean women with RA reported patterns of diagnostic delay, shifts in disclosure strategies with childbirth as the turning point, and maternal role conflict as their illness experiences. Two novel concepts, ‘forced disclosure’ and ‘legitimised vulnerability’, were derived by extending stigma management and disclosure theory and drawing on the candidacy framework in a collectivist cultural context.

### 4.1 Diagnostic Delay and Biographical Disruption

Participants experienced a mean diagnostic delay of 11.75 months (range: 6–24 months). This delay was shorter than the 17.8 months reported in a Pakistani cohort [34]. Qualitative research with Canadian women with inflammatory arthritis has likewise documented diagnostic delays of months to years, frequently because early symptoms were attributed to carpal tunnel syndrome, pregnancy, or osteoarthritis [35]. The relatively short delay observed in this study may reflect South Korea's high healthcare accessibility. However, the increased risk of diagnostic delay among women reported by Khan et al. was also evident in this study [34]. Notably, the 11.75‐month delay far exceeds the EULAR recommendation of rheumatology referral within 6 weeks of symptom onset and DMARD initiation within 3 months—the early‐RA ‘window of opportunity’—approximately an eightfold and fourfold overrun, respectively [35]. Participants attributed their symptoms to carpal tunnel syndrome or postpartum changes, consistent with patterns reported among Western female patients [36]. However, Korean participants additionally experienced self‐blame for ‘failing to properly manage postpartum recovery’. This reflects the traditional Korean health belief that inadequate postpartum care leads to physical symptoms and extends Bury's concept of biographical disruption to a collectivist cultural context [16].

### 4.2 Shifts in Disclosure Strategies With Childbirth as the Turning Point

Participants' illness disclosure strategies can be interpreted through Goffman's stigma management theory [20]. Subsequent models of health‐related stigma have developed from this framework [21]. Lempp et al. reported that RA leads to fundamental changes in self‐concept, social roles and future plans [37]. In this study, differentiated disclosure strategies, with childbirth as a turning point, represent one concrete manifestation of such identity renegotiation.

Before childbirth, participants concealed medication use through ‘passing’ strategies, behaving as though they did not have an illness. This suggests that, for unmarried participants, concealment was driven less by the illness itself than by the burden of taking medication that can cause foetal malformation and by anticipated judgement from others about this fact. Such anticipated stigma operated first on the stability of the romantic relationship—fearing that a partner's feelings might change—and secondarily on the possibility that the partner's family might treat teratogenic medication use as grounds to oppose the marriage. Underlying both was doubt about the woman's capacity to bear a healthy child. Although voluntary childlessness is increasingly accepted in Korean society, being perceived as unable to bear a healthy child—rather than choosing not to—was experienced as a devaluation of marriageability. This reflects how the ability to have a healthy child remains a core element of socially expected female identity during the childbearing years. After childbirth, participants shifted to ‘selective disclosure’, revealing the illness only to those necessary for securing childcare support. This shift was also influenced by participants' judgement that, as they no longer intended to have further children, they no longer needed to conceal taking medications that could be harmful to a foetus. Among participants in their 30s who were raising young children, disclosure was further facilitated by a sympathetic atmosphere in which their joint symptoms could be attributed to the physical demands of childcare, so that the onset or aggravation of RA was readily met with understanding rather than suspicion. By their 40s, they moved toward ‘voluntary disclosure’, revealing their condition by drawing on the age norm that ‘everyone experiences pain as they get older’. As they aged, it also became increasingly common for their colleagues to develop long‐term conditions of their own; against this backdrop, participants came to regard their RA as one of many long‐term illnesses, which lessened the sense that it was an exceptional condition or that they were uniquely affected.

Beyond childbirth, the workplace environment also shaped disclosure strategies. Disclosure was more readily supported in workplaces where colleagues themselves managed long‐term conditions, whereas a participant with seronegative RA, excluded from the Catastrophic Illness Benefit Programme, felt unrecognised as a ‘legitimate patient’ and more reluctant to disclose. These cases indicate that disclosure is shaped not only by life‐course stage but also by workplace context and institutional recognition [38].

### 4.3 Conflict Between Maternal Role and Disease Management

The conflict participants experienced between discontinuing teratogenic medication and pursuing pregnancy reflects a genuine clinical dilemma: the 2020 ACR reproductive health guideline strongly recommends discontinuing methotrexate at least 3 months before conception [39], which carries a risk of increased disease activity.

Strong maternal role expectations in Korean society led participants to internalise childrearing difficulties resulting from physical limitations as ‘not being qualified to be a mother’. Williams et al. reported that women with autoimmune rheumatic diseases experience guilt and shame arising from a discrepancy between ‘the current self’ and ‘the self who should be as a mother’, reflecting the perceived failure to meet social expectations of a ‘good mother’ [40]. The present participants' expressions of ‘feeling like I'm not qualified to be a mother’ indicate that this self‐discrepancy also manifests within the Korean cultural context.

Participants developed alternative parenting strategies to compensate for physical limitations. For example, those who found holding their children difficult maintained emotional closeness by reading beside them or talking with them frequently. This aligns with the ‘adjusting parenting to circumstances’ strategy identified among Australian mothers with RA [41] and can be understood in relation to Bury's concept of ‘biographical reconstruction’ [16].

### 4.4 Fatigue Management Strategies and Reconstruction of Normality

Participants' fatigue self‐management—including energy pacing, the use of smart home appliances, and proactive energy allocation—reflected autonomous efforts to manage limited physical resources efficiently.

These findings are consistent with the ‘plan, prioritise, pace and rest’ strategies identified by Primdahl et al. [42], suggesting that fatigue management strategies among patients with RA transcend cultural contexts. Participants with longer disease duration demonstrated self‐regulatory competence, expressed in statements such as ‘Now I know the signals my body sends’ (Participant 7). This pattern can be understood within the framework of the ‘self‐efficacy’ concept proposed by Lorig and Holman [43].

In their qualitative analysis of self‐management in RA, Donnelly et al. identified ‘renegotiating dimensions of the self’ as a core theme [5]. The proactive energy allocation observed in this study—completing tasks in advance on days when feeling well—represents a concrete manifestation of such self‐renegotiation. However, Korean participants tended not to share these strategies with family members, reflecting a collectivist psychological orientation characterised by a desire ‘not to be a burden’. Beyond avoiding dependence, participants also strove to carry out their ordinary daily tasks at work and at home, even if imperfectly, continuing to do what their everyday lives required even on days when they could not manage everything. This effort reflects their broader work of constructing and maintaining normality, the overarching theme of this study.

### 4.5 Cultural Specificity of Support Systems and Institutional Blind Spots

Participants' support systems demonstrated clear cultural specificity. Most participants received practical care support from their mothers and maternal family members alongside spousal support, reflecting activation of extended family networks. However, some experienced spousal ‘compassion fatigue’, with partners becoming emotionally distant over time, highlighting a need for nursing interventions addressing spousal support in long‐term illness. Institutionally, most participants were receiving DMARD therapy. Under South Korea's Catastrophic Illness Benefit Programme, patients pay only 10% of covered RA‐related healthcare costs. This indicates that universal health insurance enhances treatment accessibility for patients with RA. However, participants with seronegative RA (2) experienced institutional non‐recognition, because they were excluded from the Catastrophic Illness Benefit Programme despite similar symptoms and treatment. This blind spot underscores the need to re‐evaluate programme eligibility criteria based on serological status.

### 4.6 Theoretical Contribution of Novel Concepts

This study derived two novel concepts. First, ‘forced disclosure’ captures situations in which illness information is exposed without the individual's will. This concept extends Goffman's information control theory by systematising situations of ‘control failure’ among individuals with invisible illnesses [20]. It draws on a framework that informs current research on concealable stigmatised identities and health‐related stigma [22]. Contemporary disclosure scholarship, including the Disclosure Processes Model [44], theorises disclosure as a strategic process; yet these models almost uniformly assume that disclosure is volitional, occurring at the discretion of the individual [45]. ‘Forced disclosure’ identifies the boundary case that remains largely untheorised: involuntary revelation produced by the structural demands of long‐term treatment. Its operational definition includes four elements: (1) loss of information control, (2) disclosure despite preference for concealment, (3) external triggering factors and (4) psychological experience of reduced agency over disclosure. Within the contemporary stigma scholarship synthesised by the Lancet Commission, such involuntary revelation reflects the interplay of anticipated and structural stigma; although that synthesis is grounded in mental health, its constructs have been adapted directly to RA and to long‐term physical illness more broadly, providing a conceptual bridge to the present findings [46, 47, 48].

This concept is distinct from related constructs in the existing literature. While Charmaz's ‘dilemma of disclosure’ focuses on the difficulty of deciding whether to disclose [49], forced disclosure refers to situations in which the choice itself is removed. Ostuzzi et al. reported that illness disclosure in rheumatic conditions is primarily left to the individual and is shaped chiefly by psychological and social support [50]. By contrast, forced disclosure in this study describes unintentional exposure to illness due to external factors such as hospital visits and symptom flares. A frequent trigger was the obligation to give a reason when taking annual or sick leave for hospital visits, which compelled employed participants to reveal their condition to supervisors even when they wished to keep it private, regardless of age. Flick and Röhnsch reported that young adults with long‐term illness in Germany came to speak openly about their condition once they had accepted it as part of their identity, with several describing disclosure as empowering [51]. This contrasts with the pattern observed in this study in which participants preferred concealment. This difference can be explained by cultural context. In Western individualist cultures, disclosure is positively framed as ‘showing who you truly are’, whereas in Korea, personal illness may affect family reputation, increasing the psychological burden of disclosure.

Second, ‘legitimised vulnerability’ captures situations in which long‐term illness—usually concealed due to stigma—is disclosed after childbirth, such that openly revealing the illness in turn makes it possible to request and receive help legitimately. The sick role concept originally proposed by Parsons holds that acutely ill individuals are granted temporary exemption and support on the expectation of recovery, whereas those with long‐term conditions, whose illness does not resolve, are less readily granted such legitimacy [23]. This concept can be situated within the candidacy framework, which conceptualises eligibility for care as continually negotiated rather than ascribed. Recent work has applied this framework to RA specifically, theorising early‐adulthood diagnosis as an ‘untimely illness’ whose age incongruity itself complicates recognition [52]. This builds on earlier reworkings of the sick role for long‐term conditions, such as the ‘chronic sick role’ [53]. Extending this line of work, the present study found that participants no longer concealed their illness after childbirth; by framing the same symptoms in terms of child‐related care needs—an extension of the socially valued role of motherhood—they were able to request and receive help legitimately rather than being dismissed. The candidacy‐related interpretation of seronegative exclusion, however, rests on only two participants and should be regarded as provisional. While Bury's concept of biographical disruption captures life destabilisation by illness, legitimised vulnerability captures the process of becoming able to receive help by disclosing illness after childbirth [16]. These accounts may also be read through the lens of narrative medicine, in which ill persons re‐story their experience to restore biographical coherence; participants' re‐framing of illness through the maternal role represents a form of narrative reconstruction that renders vulnerability socially legitimate. This re‐storying connects the present findings to a developing lineage that extends Bury's biographical disruption through Williams's notion of narrative reconstruction, in which ill persons rebuild a coherent account of self and illness. Viewed in this light, ‘legitimised vulnerability’ is not only a candidacy‐based renegotiation of eligibility for care but also a narrative accomplishment: by reframing illness through the socially valued maternal role, participants restored both social recognition and biographical continuity [54, 55].

### 4.7 Cultural Context and Transferability

This study specifically examined how Korea's collectivist culture [56] influences the illness experiences of RA patients. Goffman's stigma management theory was developed in Western individualist contexts [20], which conceptualises illness disclosure or concealment as an individual strategic choice. However, participants in this study did not experience disclosure as a purely individual decision. Instead, they feared that disclosure would lead them to be devalued as ‘a sick person’ or ‘a weak person’, which shaped their disclosure strategies. Consistent with recent reconceptualisations of disruption as non‐linear, participants' trajectories did not follow a single onset‐to‐adaptation path but shifted between concealment and disclosure across life stages—concealing at diagnosis, disclosing selectively after childbirth, and openly in their 40s—echoing the ‘biographical oscillation’ described among young East Asian women, but organised here around childbirth as the pivotal axis [19]. These findings can be understood at three levels. Biographical disruption and fatigue self‐management appear broadly universal among people with RA, consistent with cross‐cultural syntheses of RA fatigue self‐management; [42] the relational framing of disclosure may be shared across East Asian collectivist societies, paralleling family‐mediated disclosure reported among young East Asian women [19] and reliance on the maternal family for care. By contrast, the role of the Catastrophic Illness Benefit Programme in defining ‘legitimate patient’ status, and the link between illness concealment and marriageability, appear more specifically Korean. Distinguishing these levels clarifies the potential transferability of the findings beyond the Korean context.

### 4.8 Strengths and Limitations

A notable methodological feature of this study is its anchoring of the foundational frameworks of Bury, Goffman and Parsons in their contemporary developments—including the candidacy framework and current models of disclosure—to derive two novel concepts: ‘forced disclosure’ and ‘legitimised vulnerability’. No systematic differences in interview duration or thematic content were observed between in‐person (*n* = 10) and telephone (*n* = 10) interviews. Interestingly, telephone participants appeared to provide more detailed responses to emotionally sensitive questions regarding the blow to their self‐esteem from illness‐related career loss and keeping their illness secret from their boyfriend and the people around him. This may reflect the perceived anonymity afforded by telephone interviews, facilitating disclosure of stigmatised experiences, although this observation was not systematically measured. This pattern is consistent with methodological literature suggesting that telephone interviews can facilitate disclosure of sensitive topics [31]. Offering interview mode choice based on participant preference may have enhanced comfort and willingness to share sensitive information. Regional diversity was also enhanced by including participants from Seoul as well as Gyeonggi and Gyeongsang provinces. However, this study has some limitations. Experiences from island and rural mountainous regions were not captured, and the cross‐sectional design prevented examination of changes over time. In addition, as only two participants had seronegative RA, findings concerning this subgroup—such as the link between seronegative status, exclusion from institutional benefits, and barriers to disclosure—should be interpreted with caution and warrant confirmation in larger samples. Accordingly, the candidacy‐based interpretation of seronegative exclusion is offered as a hypothesis for future investigation rather than as an established finding.

### 4.9 Recommendations for Future Research

The concepts of ‘forced disclosure’ and ‘legitimised vulnerability’ remain at a preliminary conceptualisation stage and require validation across diverse long‐term illness populations (e.g., ankylosing spondylitis, lupus and multiple sclerosis) and cultural contexts (e.g., East Asia, the Middle East, South America and other collectivist regions). In addition, quantitative instrument development and validation studies are needed to operationalise these concepts. Longitudinal studies examining changes in disclosure strategies across life stages are also warranted. Future mixed‐methods studies could explore interaction effects between life‐cycle factors (e.g., childbirth status and age group) and contextual factors (e.g., workplace support and institutional inclusiveness) to model disclosure decision‐making mechanisms more precisely.

### 4.10 Implications for Nursing Practice and Policy

Nurses in primary care facilities and obstetrics/gynaecology and orthopaedics departments of secondary hospitals play a critical role in reducing the mean 11.75‐month diagnostic delay. Participants initially visited community primary care or orthopaedic clinics rather than rheumatology departments at symptom onset. Based on the ACR/EULAR 2010 classification criteria, orthopaedic nurses may wish to consider referral to rheumatology when morning stiffness persists beyond 6 weeks, symmetric swelling affects three or more joints, and fatigue is present. Obstetrics and gynaecology nurses may consider recommending further evaluation for inflammatory arthritis when postpartum joint pain persists beyond 4 weeks.

Nursing interventions may be differentiated based on life‐course stage, with pregnancy‐ and childbirth‐related counselling for pre‐childbirth patients and support for building support systems that utilise ‘legitimised vulnerability’ for post‐childbirth patients [57]. From a policy perspective, it is vital to address the exclusion of patients with seronegative RA from the Catastrophic Illness Benefit Programme and develop policies for reasonable workplace accommodation for individuals with long‐term illness.

## 5 Conclusion

The illness experiences of young women with RA are complex, shaped by intersecting life‐course, cultural and gender contexts beyond the biomedical dimension. The overarching theme ‘Constructing normality between uncertainty and life‐course tasks’ shows that participants are not merely managing illness but continually renegotiating their sense of self and social identities to establish personal normality.

A key finding of this study is that childbirth functions as a turning point in illness disclosure strategies. Before childbirth, participants controlled discreditable information through active concealment, whereas after childbirth, they gained legitimacy for help‐seeking through the socially valued role of motherhood. These life stage‐specific experiences highlight the need for nursing approaches tailored to patients' life‐course stages.

The newly derived concepts of ‘forced disclosure’ and ‘legitimised vulnerability’ advance nursing knowledge by extending stigma management theory, disclosure theory and the candidacy framework into a collectivist cultural context. Defined through four operational components each, these concepts provide a basis for future measurement development. Accordingly, nurses could implement life‐cycle‐tailored interventions, and policymakers may wish to consider addressing the exclusion of patients with seronegative RA from the Catastrophic Illness Benefit Programme and ensure reasonable workplace accommodation for individuals with long‐term illness.

## Author Contributions

**Wonhee Baek:** conceptualisation, methodology, formal analysis, writing – review and editing, funding acquisition, supervision, writing – original draft. **Hyunjung Doo:** conceptualisation, methodology, formal analysis, investigation, writing – review and editing; writing – original draft.

## Disclosure

The authors have nothing to report.

## Ethics Statement

This study received approval from the Gyeongsang National University Institutional Review Board (IRB No. GIRB‐G25‐NY‐0126; Approval date: 5 November 2025). Written consent was obtained after participants were informed of the study purpose, procedures, voluntary participation and withdrawal rights, and confidentiality protections, with separate consent for interview recording.

## Conflicts of Interest

The authors declare no conflicts of interest.

## Data Availability

The data that support the findings of this study are available on request from the corresponding author. The data are not publicly available due to privacy or ethical restrictions.
